# How Applicable Is the Single-Dose AMBITION Regimen for Human Immunodeficiency Virus–Associated Cryptococcal Meningitis to High-Income Settings?

**DOI:** 10.1093/cid/ciac792

**Published:** 2022-09-27

**Authors:** Thomas S Harrison, David S Lawrence, Henry C Mwandumba, David R Boulware, Mina C Hosseinipour, Olivier Lortholary, Graeme Meintjes, Mosepele Mosepele, Joseph N Jarvis

**Affiliations:** Institute of Infection and Immunity, St George's University London, London, United Kingdom; Clinical Academic Group in Infection and Immunity, St George's University Hospitals NHS Foundation Trust, London, United Kingdom; Medical Research Council Centre for Medical Mycology, University of Exeter, Exeter, United Kingdom; Department of Clinical Research, Faculty of Infectious and Tropical Diseases, London School of Hygiene and Tropical Medicine, London, United Kingdom; Botswana Harvard AIDS Institute Partnership, Gaborone, Botswana; Clinical Sciences Department, Liverpool School of Tropical Medicine, Liverpool, United Kingdom; Malawi-Liverpool-Wellcome Clinical Research Programme, Blantyre, Malawi; Department of Medicine, Kamuzu University of Health Sciences, Blantyre, Malawi; Infectious Diseases Institute, College of Health Sciences, Makerere University, Kampala, Uganda; Division of Infectious Diseases and International Medicine, University of Minnesota, Minneapolis, Minnesota, USA; Lilongwe Medical Relief Trust (University of North Carolina Project), Lilongwe, Malawi; Department of Medicine, University of North Carolina, Chapel Hill, North Carolina, USA; Institut Pasteur, National Center for Scientific Research, Molecular Mycology Unit and National Reference Center for Invasive Mycoses and Antifungals, Unités Mixtes de Recherche 2000, Paris, France; Université de Paris-Cité, Necker Pasteur Center for Infectious Diseases and Tropical Medicine, Hôpital Necker Enfants Malades, Assistance Publique–Hôpitaux de Paris, IHU Imagine, Paris, France; Wellcome Centre for Infectious Diseases Research in Africa, Institute of Infectious Disease and Molecular Medicine, Cape Town, South Africa; Department of Medicine, University of Cape Town, Cape Town, South Africa; Botswana Harvard AIDS Institute Partnership, Gaborone, Botswana; Department of Internal Medicine, University of Botswana, Gaborone, Botswana; Department of Clinical Research, Faculty of Infectious and Tropical Diseases, London School of Hygiene and Tropical Medicine, London, United Kingdom; Botswana Harvard AIDS Institute Partnership, Gaborone, Botswana

**Keywords:** cryptococcal meningitis, HIV, amphotericin B, fluconazole, flucytosine

## Abstract

The AmBisome Therapy Induction Optimization (AMBITION-cm) trial, conducted in eastern and southern Africa, showed that a single, high dose (10 mg/kg) of liposomal amphotericin B, given with an oral backbone of fluconazole and flucytosine, was noninferior to the World Health Organization (WHO)–recommended regimen of 7 days of amphotericin B deoxycholate plus flucytosine for treatment of human immunodeficiency virus (HIV)–associated cryptococcal meningitis and has been incorporated into WHO treatment guidelines. We believe that the trial also has important implications for the treatment of HIV-associated cryptococcal meningitis in high-income settings. We advance the arguments, supported by evidence where available, that the AMBITION-cm trial regimen is likely to be as fungicidal as the currently recommended 14-day liposomal amphotericin–based treatments, better tolerated with fewer adverse effects, and confer significant economic and practical benefits and, therefore, should be included as a treatment option in guidance for HIV-associated cryptococcal treatment in high-income settings.

## Teaser

The AMBITION-cm trial showed that a single, high dose of liposomal amphotericin B, given with oral fluconazole and flucytosine, is an effective treatment for human immunodeficiency virus–associated cryptococcal meningitis. We argue that this is an appropriate treatment option for high-income settings.

Human immunodeficiency virus–associated cryptococcal meningitis remains a significant driver of AIDS-related mortality, causing about 15% of all AIDS-related deaths. The greatest burden of disease is found in sub-Saharan Africa [1], primarily due to the persistent burden of advanced HIV disease despite widespread access to antiretroviral therapy [2]. Given the distribution of global disease burden, the vast majority of recent clinical research that guides cryptococcal management in people with HIV has been generated in low- and middle-income countries (LMICs) [3].

Although the disease burden has lessened in high-income countries, HIV-related cryptococcosis still occurs and mortality is still substantial [4], with an estimated 7400 cases and 2000 deaths annually across Europe and North America [1]. In this viewpoint, we present an overview of the findings of the recent AmBisome Therapy Induction Optimization (AMBITION) trial [5] and discuss their applicability to high-income settings. Data generated in LMICs have historically been overlooked in the development guidelines for of high-income countries. It is challenging to compare contexts, particularly when control regimens used in LMIC trials differ from the standard of care in high-income countries and when the ability to monitor and manage other HIV- and treatment-related complications varies, such that simple comparison of reported mortalities in high-income and LMIC studies is inappropriate. Nevertheless, we argue that recent, high-quality data for novel treatment approaches from large, multisite, randomized, controlled trials provide critical insights into drug action and toxicity and options for treatment that are universally applicable and should therefore be considered in high-income settings. We cover only HIV-associated cryptococcal meningitis; treatment of other risk groups requires specific studies.

##  

### Short-Course Amphotericin-based Treatment for Cryptococcal Meningitis

A program of clinical trials across sub-Saharan Africa was initiated in 2004. The aim was to develop and test new antifungal regimens, based on current drugs, that would be safer and more sustainable than the international standard of 2 weeks of amphotericin plus flucytosine, established by the AIDS Clinical Trials Group trial of van der Horst and colleagues [6], and also be more effective than widely available and used fluconazole monotherapy [7]. Based on a series of promising phase 2 studies [8–10], the Advancing Cryptococcal Treatment in Africa (ACTA) trial recruited 722 people with HIV-related cryptococcal meningitis who were randomized to 1 of 5 arms: oral combination therapy with high-dose fluconazole (1200 mg/d) plus flucytosine, 1 week of amphotericin deoxycholate (1 mg/kg/d) with either fluconazole (1200 mg/d) or flucytosine (100 mg/kg/d), or the established standard of 2 weeks of amphotericin deoxycholate, again with either fluconazole or flucytosine [11]. One week of amphotericin was noninferior to treatment for 2 weeks in terms of all-cause mortality through 10 weeks (hazard ratio [HR], 0.89; 95% confidence interval [CI], .66–1.21). In addition, those randomized to 1 week of amphotericin plus flucytosine experienced the lowest 10-week mortality when compared with all other regimens, including 14 days of the same amphotericin plus flucytosine therapy (24% vs 38%; HR, 0.56; 95% CI, .35–.91). The results reflect an optimal balance between fungicidal activity and toxicity with the 1-week regimen. The shorter course of amphotericin significantly reduced amphotericin-related toxicities, particularly anemia and renal impairment, without a reduction in fungicidal activity, probably due to the long half-life of amphotericin. Flucytosine was the best partner drug with amphotericin B, associated with reduced mortality and enhanced fungicidal activity. The oral combination arm had the fewest side effects and was the second best-performing regimen overall, despite less rapid fungicidal activity. As a result, in 2018, the WHO recommended 1 week of amphotericin plus flucytosine followed by 7 days of fluconazole 1200 mg/d as first-line therapy [12]. In addition, the oral combination arm was recommended if amphotericin was unavailable.

Subsequently, advocacy efforts and support from Unitaid and partners has led to increasing availability of more affordable generic flucytosine, and results of implementation of the 1-week amphotericin plus flucytosine regimen have mirrored the mortality reduction seen in the ACTA trial, with in-hospital mortality in South Africa reduced from 37% (based largely on the prior standard there of 2 weeks of amphotericin plus fluconazole) to 24% [13].

However, even a 1-week course of amphotericin deoxycholate has significant toxicities [11], which prompted work to determine if novel, short-course treatment with liposomal amphotericin (AmBisome, Gilead Sciences, Foster City, CA) could be clinically efficacious, safe, and cost-effective. Proof of concept for a single, high dose of AmBisome was established in visceral leishmaniasis [14]. In addition, AmBisome has a very long half-life in brain tissue in animal models [15].

### Single High-Dose Liposomal Amphotericin-based Therapy

The AMBITION phase 2 trial was a multisite, randomized, controlled trial with the objective of finding the optimal high-dose, short-course AmBisome dosing strategy for cryptococcal meningitis [18]. Early fungicidal activity (EFA) was the primary end point. While EFA, the rate of fall in cryptococcal colony-forming units per milliliter of cerebral spinal fluid (CSF; derived from serial lumbar punctures and quantitative CSF cultures) per day, is not a perfect surrogate and does not capture issues of toxicity, it is independently associated with clinical outcome and is a quantitative metric of antifungal activity at the site of infection in humans [17, 16]. The trial had 4 arms: AmBisome 10 mg/kg/d on day 1 (single dose), AmBisome 10 mg/kg/d on day 1 and 5 mg/kg/d on day 3 (2 doses), AmBisome 10 mg/kg/d on day 1 and 5 mg/kg/d on days 3 and 7 (3 doses), and AmBisome 3 mg/kg/d for 14 days (control). All patients also received fluconazole 1200 mg/d for 14 days.

Eighty participants were enrolled before the study was stopped on recommendation of the independent data and safety monitoring committee. The antifungal activity was similar across the 3 short-course, high-dose AmBisome arms, which were all noninferior to the control 14-day regimen (EFA of single dose: −0.52 log_10_ CFU/mL/d and standard deviation [SD] 0.35 vs control: −0.41 log_10_ CFU/mL/d and SD 0.11), with no suggestion of a dose response with additional doses and no safety concerns [16]. The single-dose regimen was therefore taken forward to phase 3.

At that time, the ACTA trial results became available showing the superiority of flucytosine as a partner drug with amphotericin. There were, however, concerns that if we simply switched to a single-dose AmBisome plus flucytosine combination, low levels of AmBisome could effectively result in flucytosine monotherapy toward the end of the induction period, risking the development of flucytosine resistance given its low barrier to resistance. Adding single-dose AmBisome to the optimized oral backbone of high-dose fluconazole plus flucytosine, which even on its own performed well in the ACTA trial, would protect flucytosine while giving a needed amphotericin-related fungicidal boost to the oral regimen. In addition, prior phase 2 studies [10] supported earlier animal model work [19] that showed that when higher, more effective doses of fluconazole are used, the triple combination of amphotericin, flucytosine, and fluconazole is associated with the most rapid fungicidal activity (in contrast to earlier results using lower fluconazole doses [20]). The intervention thus brought together the strength of the oral combination arm observed in the ACTA trial and added the single, high dose of AmBisome shown in the AMBITION phase 2 trial to be safe and the most practical and efficient means to deliver liposomal amphotericin.

### AMBITION-cm Trial

The AMBITION phase 3 trial was a noninferiority, randomized, controlled trial of a single, high-dose of AmBisome given with 14 days of flucytosine and fluconazole in comparison with the WHO standard of care as previously defined: 7 days amphotericin deoxycholate plus flucytosine, followed by 7 days of fluconazole [5, 12]. The trial recruited 844 participants from 8 hospitals in 5 countries: Botswana, Malawi, South Africa, Uganda, and Zimbabwe. A total of 814 participants were included in intention-to-treat analysis, 407 in each arm, and no participants were lost to follow-up. At enrollment, the median CD4 count was 27 cells/mm^3^, and 28.5% of participants had abnormal mental status, indicating severe disease. Ten-week mortality was 24.8% (101 of 407; 95% CI, 20.7%–29.3%) in the AmBisome arm and 28.7% (117 of 407; 95% CI, 24.4%–33.4%) in the control arm. The absolute difference in 10-week mortality risk between the AmBisome arm and control arm was −3.9% with an upper-limit 1-sided 95% CI of 1.2%, well below the prespecified 10% noninferiority margin. When adjusted for factors associated with mortality, the AmBisome regimen was found to be just superior at 10 weeks. The mean rate of fungal clearance from the CSF was −0.40 log_10_ CFU/mL/d in the AmBisome group and −0.42 log_10_ CFU/mL/d in the control group with no significant difference between arms [5].

In addition, the AmBisome regimen was associated with significantly fewer adverse events including anemia, thrombophlebitis, and electrolyte abnormalities. Grade 3 or 4 anemia developed in 13.3% of participants on AmBisome compared with 39.1% in the control group (*P* < .001) [5]. The mean decrease in hemoglobin over the first week was 0.3 g/dL for the AmBisome group and 1.9 g/dL for the control group (*P* < .001); 7.6% of participants on AmBisome received a blood transfusion compared with 18.0% for the control group. The mean increase in creatinine from baseline to day 7 was 20.2% for the AmBisome group and 49.7% for the control group (*P* < .001). Thrombophlebitis that required antibiotic therapy occurred in 1.9% of participants on AmBisome and 6.7% for the control group (*P* = .001). There was a low frequency of grade 4 thrombocytopenia, neutropenia, and elevated alanine aminotransferase in both AmBisome and control groups. The results prompted the WHO to update their guidance to recommend the single 10-mg/kg liposomal amphotericin–based regimen as the preferred regimen [21]. Implementation efforts in LMICs are already underway, supported by Unitaid, the Centers for Disease Control and Prevention, Clinton Health Access Initiative, Médecins Sans Frontières, and others. Gilead has reaffirmed their commitment to not-for-profit LMIC pricing for AmBisome for cryptococcal meningitis [22].

### Use of the AMBITION Regimen in High-Income Settings

What about use of the AMBITION regimen in high-income settings? Are patients living with HIV in high-income countries now actually at risk of being “left behind,” with unnecessarily long and toxic 2-week courses of daily liposomal amphotericin or even amphotericin deoxycholate? The recommended first-line induction regimen in high-income settings is liposomal amphotericin at 3–4 mg/kg plus flucytosine 100 mg/kg/d for 14 days, a recommendation that is consistent with those from the Infectious Diseases Society of America, British HIV Association, and European AIDS Clinical Society [23–26]. These guidelines are based on the van der Horst trial of 2 weeks of amphotericin deoxycholate plus flucytosine [6] and a subsequent transition over time from conventional amphotericin deoxycholate to the liposomal formulation, based on the study of Hamill and colleagues who compared the liposomal and deoxycholate formulations given as monotherapy [16]. To date, no randomized, controlled trials have tested the 2-week liposomal amphotericin plus flucytosine treatment regimen recommended in these guidelines.

#### Fungicidal Activity

In terms of fungicidal activity, the AMBITION phase 3 trial demonstrated that the EFA of the single, high-dose AmBisome regimen was no different from that achieved with 7 days of amphotericin deoxycholate–based treatment. Also, the AMBITION phase 2 data show that the single, high-dose regimen is noninferior and may be marginally superior in EFA to standard daily AmBisome dosing for 14 days. Although the number of patients treated was small, the EFA was more rapid than control daily dosing across the 3 intermittent dosing arms (−0.52, −0.47, and −0.54 for 1, 2, and 3 doses, respectively, compared with daily −0.41 log_10_ CFU/mL/d [16]), perhaps due to more rapid loading of brain compartments with the initial 10-mg/kg dose on day 1. In addition, the effect of the single-dose regimen is durable. In the AMBITION trial, no culture-positive relapses occurred within the 10-week follow-up period in the 407 participants treated [5]. This is despite the fact that the trial included many patients with severe disease and heavy organism load and participants with, in general, higher fungal burdens than usually seen in high-income settings [5, 6].

Hamill et al’s trial is the only one to provide data on the sterilizing effect of daily AmBisome for 14 days in high-income settings [27]. This was a 3-arm comparison of amphotericin deoxycholate 0.7 mg/kg, AmBisome 3 mg/kg, and AmBisome 6 mg/kg in North America, with the aim of administering a full, uninterrupted 14-day course. A minimum of 11 days was required; in participants with delayed improvement, treatment was continued for up to 21 days. The primary outcome was CSF sterility at 2 weeks. Eighty-six participants were randomized to 3 mg/kg AmBisome, 94 to 6 mg/kg/d AmBisome, and 87 to amphotericin deoxycholate. Of note, a number of participants did not complete the study for reasons that included adverse events, lack of efficacy, loss to follow-up, and physician decision. Repeat CSF cultures at 14 days were negative in 58% of evaluable patients (positive baseline culture and at least 1 follow-up culture) who received AmBisome 3 mg/kg, 48% with AmBisome 6 mg/kg, and 48% with amphotericin deoxycholate. By 10 weeks, the percentage evaluable with negative CSF cultures was 60%, 71%, and 79%, respectively [27]. This compares with 77% (255 of 332) CSF culture conversion at 2 weeks for the single-dose AmBisome regimen in AMBITION where all survivors had a day 14 lumbar puncture [5].

**Figure 1.**
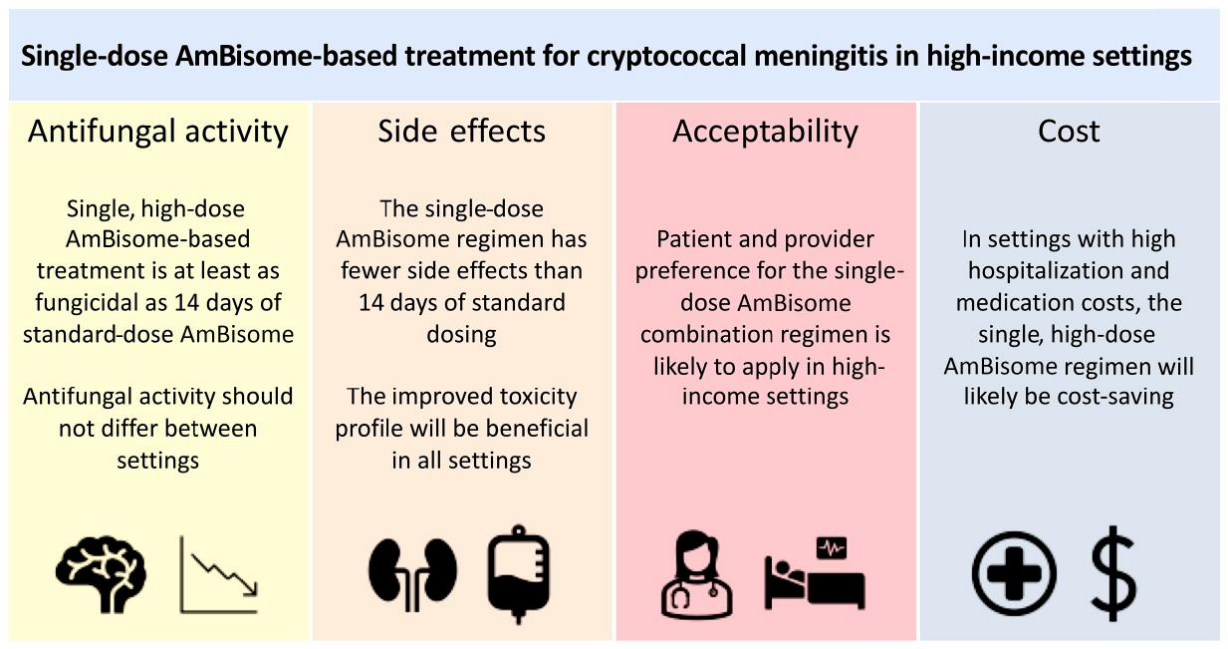
The use of single-dose AmBisome based treatment for cryptococcal meningitis in high-income settings.

While there is no large, randomized trial comparison, it is plausible that daily 3–4 mg/kg/d AmBisome is less active in terms of EFA than either amphotericin deoxycholate at 1 mg/kg/d or the AMBITION single, high-dose regimen, with no data to suggest that 3–4 mg/kg/d AmBisome would be more fungicidal. We would contend that overall the evidence suggests that the AMBITION triple-therapy regimen would have at least equivalent fungicidal activity as 14 days of daily 3–4 mg/kg AmBisome plus flucytosine and should not be regarded as a compromise regimen of interest only in resource-limited settings in terms of antifungal effect (Figure 1).

#### Clinical Efficacy

It is difficult to generalize that AmBisome is equally effective but safer than amphotericin deoxycholate; it depends on the dose of both drugs. Hamill et al [27] used amphotericin deoxycholate at 0.7 mg/kg/d, and we know from LMIC data that 1 mg/kg/d has greater antifungal activity [28]. Based on the Hamill et al study comparing Ambisome with 0.7 mg/kg/d deoxycholate [27], the US Food and Drug Administration (FDA) approved only the 6-mg/kg/d dose of AmBisome [29]. In the FDA analysis, combined clinical success and culture conversion by 10 weeks in those with a positive baseline culture was 37%, 49%, and 53% for AmBisome 3 mg/kg/d, AmBisome 6 mg/kg/d, and amphotericin deoxycholate, respectively [29]. However, with 6 mg/kg/d, adverse events were comparable to those with amphotericin deoxycholate [27] and costs were increased substantially.

Mortality in the van der Horst trial was 5.5% at 2 weeks and 3.9% at between 2 and 10 weeks, and it is sometimes assumed therefore that 10-week mortality was 9.4%. However, the trial was conducted in 2 stages, and only participants who responded to treatment at 2 weeks were continued in the trial and re-randomized to fluconazole vs itraconazole for consolidation treatment [6]. Of 381 participants initially randomized, 21 died in the first 2 weeks and 12 died at between 2 and 10 weeks in the second step. Of 360 survivors at 2 weeks, 54 (who were not stable or had not improved and were likely to have poor outcomes) were not followed up; 7 were known to have died, but outcomes for the other 47 are unknown (van der Horst, written personal communication in May 2010). Thus, 10-week mortality was actually up to 23% (87 of 361), depending on how many of these participants died before 10 weeks. In addition, there was no next-of-kin consent and the exclusion criteria were more extensive than in the ACTA and AMBITION trials. In those trials, patients were not excluded on the basis of markers of severity and next-of-kin could consent for confused patients and patients with a reduced conscious level (in AMBITION, 10-week mortality with the AmBisome regimen for those with Glasgow Coma Score 15 was 16.8%). Cohort data from high-income settings showed 70- to 90-day mortalities of 15%–26% [4, 30–33]. This is, overall, lower but not so different from our latest trial results and probably driven by earlier presentation and greater ability to monitor and manage other HIV- and treatment-related complications, rather than superiority of antifungal regimen. Thus, we would argue that the AMBITION regimen should not be ruled out for high-income settings on the basis of mortality comparisons.

#### Safety

In terms of safety, the data suggest that the single-dose AmBisome regimen has advantages over current guidance. In the Hamill et al trial, 23.3% of those on 3 mg/kg/d and 41% of those on 6 mg/kg/d AmBisome developed hemoglobin levels ≤8 g/dL [27]. In the AMBITION study, the cutoff for grade 3 anemia was <9.0 g/dL in women and <8.5 g/dL in men, yet only 13.3% of those on the single-dose regimen developed this level of anemia [5]. In prior studies, both anemia and increases in creatinine have been associated with increased mortality [34]. As described above, the AMBITION regimen was similarly “clean” in terms of renal impairment, hypokalemia, and, unsurprisingly, given the need for just 1 infusion, line infections – a source of serious bacterial sepsis [35]. While consistent close monitoring and management of side effects may be more feasible in high-income settings than in LMIC settings, this does not eliminate the occurrence of serious side effects nor does it completely avoid the associated morbidity and mortality. Patients in high-income settings will also benefit from a safer regimen.

#### Cost and Acceptability

In terms of cost, convenience, and patient and provider preference, the AMBITION regimen has clear advantages. In a formal health economic analysis, the AMBITION regimen was only marginally more costly than 1 week of amphotericin deoxycholate plus flucytosine [36]. Additional comparisons are underway, but there will be very significant cost savings with the AMBITION regimen compared with 2-week liposomal amphotericin–based regimens, driven by the possibility of shorter hospitalization and a 5-fold reduction in AmBisome drug requirement (10 mg/kg total vs 49 mg/kg total for a 14-day course at 3.5 mg/kg/d). In a retrospective analysis of 24 151 patients with HIV-associated cryptococcal meningitis who were treated in the United States between 1997 and 2009, the average hospitalization cost was calculated to be $15 708 per patient [37]; these costs have increased significantly since that time [38]. In addition, a social science substudy of the AMBITION trial points to a clear preference on the part of participants and healthcare providers for the single-dose regimen [39]. Given their vulnerable status and ongoing nosocomial infection risks, patients in high-income countries with less severe disease may also welcome and benefit from the simplified delivery of treatment and the possibility of earlier discharge with the AMBITION regimen. The median duration of hospitalization in successful implementation of the 1-week amphotericin deoxycholate regimen in South Africa was 10 days [14], and discharge before day 14 could be conditional on close outpatient follow-up.

## CONCLUSIONS

There are no recent controlled trials of HIV-associated cryptococcal meningitis from high-income countries. Thirteen trials have been published between 1990 and 2010; those trials recruited 1623 patients in high-income settings from 1987 to 2007 [3]. This compares with 4275 patients recruited in LMICs up to and including the recent AMBITION trial [3]. No randomized, controlled trial data support current European and US treatment guidance, and new high-income country–only trials will be challenging due to the dispersed case burden. Future trials that incorporate new antifungal agents should include recruitment in high-income settings with local standard-of-care comparisons at those sites. Meanwhile, for high-income countries, careful evaluation of evidence from LMICs is warranted, just as physicians in LMIC settings routinely adapt evidence from high-income countries to their context.

In conclusion, there are limited comparable data from high-income countries to clearly compare the clinical, microbiological, and safety outcomes observed in the AMBITION trial with those observed in high-income settings where patients are managed with 2 weeks of liposomal amphotericin plus flucytosine. The EFA, high rates of CSF sterility at 2 weeks, and absence of relapse cases observed in the AMBITION trial indicate that the AMBITION antifungal combination is extremely effective at clearing *Cryptococcus* from the CSF. In addition, the shorter duration of intravenous treatment and the low rates of drug-related toxicity compared with clinical trial data of prolonged courses of liposomal amphotericin indicate that this is a safe and convenient treatment regimen. We would argue that the AMBITION regimen should be included as a treatment option in guidance for HIV-associated cryptococcal treatment in high-income settings. As with any new treatment, context-specific algorithms could enable optimal, safe delivery, and ongoing monitoring and evaluation of outcomes will be important.

## Notes

***Financial support*.** Funding was provided by the National Institute for Health Research through a Global Health Research Professorship (RP-2017-08-ST2-012, to J. N. J.) with aid from the UK government to support global health research.
